# Improving the management of left ventricular thrombus in a tertiary cardiology centre: a quality improvement project

**DOI:** 10.1136/bmjoq-2022-002111

**Published:** 2023-01-11

**Authors:** Nithusa Rahunathan, Ben Hurdus, Sam Straw, Hansa Iqbal, Klaus Witte, Stephen Wheatcroft

**Affiliations:** 1 Department of Cardiology, Leeds Teaching Hospitals NHS Trust, Leeds, UK; 2 Leeds Institute of Cardiovascular and Metabolic Medicine, University of Leeds, Leeds, UK

**Keywords:** Quality improvement, Clinical pharmacology, Efficiency, Organizational, Patient-centred care

## Abstract

Left ventricular (LV) thrombus is an increasingly recognised complication following anterior myocardial infarction and non-ischaemic cardiomyopathy. Whilst vitamin K antagonists (VKA) remain the only approved therapeutic option to reduce the risk of systemic thromboembolism including stroke, the off-label use of direct oral anticoagulants (DOACs) is becoming an attractive alternative.

We aimed to improve the diagnosis and management of LV thrombus at a tertiary cardiology centre using quality improvement methodology. Outcomes included increasing the use of DOACs from 25% to 70% over a period of 1 year and shorten length of time from diagnosis to repeat imaging to within 3–6 months as recommended by guidelines.

During the first Plan–Do–Study–Action (PDSA) cycle, we identified 84 patients diagnosed with LV thrombus between 1 December 2012 and 30 June 2018. The majority (74%) were prescribed VKA. Repeat imaging occurred in 89% of patients, but only 55% using the same modality. The mean duration between diagnosis and repeat imaging was 233±251 days. There were no significant differences between VKA and DOAC in terms of thrombus resolution, systemic embolisation or clinically significant bleeding. We published trust-wide guidelines on the management of LV thrombus with recommendations supporting the use of DOACs and appropriate follow-up imaging. A second PDSA cycle undertaken between 1 October 2019 and 31 March 2020 identified a further 20 patients. DOAC use increased to 70% and 70% of patients underwent follow-up imaging following a mean duration of 140±61 days, although in only 36% using the same modality.

Using quality improvement methodology, we confirmed safe and efficient use of DOAC in the setting of LV thrombus. We published trust guidelines supporting their use, which was associated with an increase in DOAC use and in earlier follow-up imaging in line with our recommendations.

**WHAT IS ALREADY KNOWN ON THIS TOPIC**

- Vitamin K antagonists (VKA) have historically been the standard of care for the management of left ventricular (LV) thrombus following myocardial infarction, direct oral anticoagulant (DOAC) use is now the treatment of choice for venothromboembolism risk reduction in atrial fibrillation, which makes it a potential attractive alternative in LV thrombus management.

**WHAT THIS STUDY ADDS**

- This study adds to the growing body of evidence that DOAC may be as least as effective and safe as VKA in the management of LV thrombus.

**HOW THIS STUDY MIGHT AFFECT RESEARCH, PRACTICE OR POLICY**

- This will add to the growing confidence clinicians have in the use of DOAC and encourage further study in to efficacy of DOAC in the management of LV thrombus particularly when compared with VKA.

## Background

Left ventricular (LV) thrombus is an increasingly recognised complication following anterior myocardial infarction (MI) and non-ischaemic cardiomyopathy. The reported incidence varies considerably, with risk factors including extent and location of infarction, use of reperfusion therapy, and the timing and modality of imaging used. Around 4% of patients who undergo primary percutaneous coronary intervention (PCI) following ST-elevation MI (STEMI) have evidence of LV thrombus on contrast or non-contrast transthoracic echocardiography, the majority of which result from anterior STEMI causing severe LV systolic dysfunction.^1 2^ Cardiac MR (CMR) imaging has greater sensitivity, with LV thrombus often discovered incidentally during imaging undertaken for other indications.^3^ LV thrombus predisposes to thromboembolic events, including stroke and is a source of potentially avoidable morbidity and mortality.^4^

The risk of systemic thromboembolism is reduced by anticoagulation, which has traditionally been provided through the use of vitamin K antagonists (VKA). Although not licensed for this indication these agents (mostly warfarin) are regarded as standard of care. Current European Society of Cardiology guidelines recommend systemic anticoagulation for at least 6 months, followed by repeat imaging to ensure thrombus resolution. However, no specific recommendations are made regarding the type of anticoagulant.^5^ The off-label use of direct oral anticoagulants (DOAC) has become an attractive alternative in this setting due to their lack of need for monitoring and dose adjustments.^6^ DOACs have become contemporary treatments for other cardiovascular conditions including non-valvular atrial fibrillation, in which their efficacy is similar to VKA, despite lower rates of bleeding.^2^ However, it is currently unclear whether DOACs have similar efficacy as VKA in terms of reducing the risk of systemic embolism including stroke and thrombus resolution in the setting of LV thrombus. Furthermore, the safety profile, specifically bleeding rates, in this setting in which patients often receive antiplatelet therapy in addition to anticoagulation is unclear.

## Problem

Although not licensed for this indication, the use of DOACs for the treatment of LV thrombus is becoming more common, despite the fact that their safety and efficacy in this setting have not been established. Furthermore, repeat imaging is often not undertaken, and moreover, even if repeat imaging is performed, it is often not within a specific time frame or the same modality as that which made the original diagnosis. We aimed to develop a sustainable intervention that would support clinicians to manage LV thrombus following anterior MI at a tertiary cardiology centre.

## Measurement

The Leeds Teaching Hospitals NHS Trust (LTHT) comprises of two teaching hospitals, providing tertiary cardiology and cardiac surgery services. LTHT provides the primary PCI service for the West Yorkshire region, which has a population of approximately 3.5 million people and has routine access to echocardiography and MRI.

The project aims were first, to establish a pathway that advocates for the role of DOACs in the management of LV thrombus, and second, to develop a sustainable intervention to standardise practice in respect to treatment modality and repeat imaging using quality improvement methodology. We anticipated that if the associated outcomes with DOACs can be demonstrated to be similar to VKA, their use might increase from approximately 25% to 70% over a period of 1 year, and that our project might reduce the interval from diagnosis to repeat imaging to 6 months in line with recommendations.

Initially, we conducted a retrospective service evaluation of patients diagnosed with LV thrombus at our institution. We assessed the modality of imaging, type of anticoagulation, thrombus resolution, thromboembolic events, major and minor bleeding, and the timing and modality of follow-up imaging. We then published and disseminated guidelines and a protocol for the management of LV thrombus at LTHT (http://lhp.leedsth.nhs.uk/detail.aspx?id=6143) with the aim of standardising practice. The protocol included advice on choice of anticoagulant, duration of treatment and the requirement for repeat imaging using the same modality.^7^

The baseline data collection included consecutive patients diagnosed with LV thrombus between 1 December 2012 and 30 June 2018. Routinely collected patient information was obtained using local electronic healthcare records. Clinical data included demographics (age, sex and self-identified ethnicity, body mass index) and comorbidities (diabetes mellitus, hypertension, atrial fibrillation, peripheral vascular disease, smoking history, history of cerebrovascular disease, previous thromboembolism). We also recorded baseline investigations including laboratory values (serum haemoglobin, platelet count, creatinine) and cardiac imaging (imaging modality, LV ejection fraction, regional wall motion abnormalities and location of thrombus). We planned to use the same methodology as our initial baseline service evaluation for collecting data after introducing our sustainable intervention.

## Design

The project team consisted of cardiologists, junior doctors, a consultant pharmacist and medical student who worked collaboratively throughout two Plan–Do–Study–Action (PDSA) cycles. The findings of the baseline data were used to develop an intervention to improve the diagnosis and management of LV thrombus within LTHT. The concept of the project was to ensure that clinicians managing these patients had an accessible and comprehensive guideline that they could reference to improve the consistency of patient care.

As an initial intervention, our findings from PDSA cycle 1 prompted us to implement trust-wide guidelines providing recommendations on choice of anticoagulation and guidance on appropriate follow-up imaging.^6^ The guidelines included recommendations on diagnosis of LV thrombus, baseline investigations, initial management, time frame to follow-up with imaging and who to contact for specialist advice. As a second intervention, these data were presented at an international conference and also published in a peer-reviewed journal, allowing clinicians to have confidence in these evidence-based recommendations that DOACs appeared to be a safe and effective choice of anticoagulation for LV thrombus.^8^

The two interventions were deemed to be the most efficient and sustainable way of improving care within the trust because they could be easily accessed. They provided the necessary information to manage non-complex cases out of hours without specialist referral and help to improve the rate of appropriate referral and follow-up. These guidelines would subsequently be re-evaluated according to our governance processes.

## Strategy

### PDSA cycle 1

The strategy for our project was to develop and implement trust guidelines to aid clinicians in managing new cases of LV thrombus. The guidelines were written and published to be easily accessible online and provide a structured approach with clarity on the therapeutic options. Initially, we thought a departmental educational meeting may be the most useful way, however, it became apparent that it would be challenging to reach all of those involved in patient care and to guarantee retention of knowledge. We also were aware that this session would need to be repeated regularly due to the rotations of junior and middle-grade clinicians both within the department and to other hospitals. Hence, we decided that a guideline would be more inclusive and effective.

The guidelines written by SW and KW included information on how LV thrombus may be detected post-MI, who to contact if picked up incidentally, and which group of patients should be screened and at which point in the course of their acute illness. Recommendations for management included timing of initiating therapy, duration of treatment, monitoring parameters and key factors to consider when choosing type of anticoagulation therapy such as renal function, body weight and concurrent antiplatelet therapy. The guidelines advised follow-up imaging with same modality as initial diagnosis and outpatient review to be arranged at about 3 months. The guidelines, which were approved by the LTHT Drugs and Therapeutics Committee and the LTHT Clinical Guidelines Group, were published on 1 October 2019 onto the LTHT intranet. These recommendations were supported by the primary data being published in a peer reviewed journal and also presented in an international conference.

### PDSA 2 cycle

Following our interventions, a further service evaluation was undertaken looking at new diagnoses of LV thrombus in LTHT from 1 October 2019 to 31 March 2020. This specific time frame was chosen as the guidelines were published on 1 October 2019, therefore, allowing us to observe whether the guidance had affected particularly the choice of anticoagulant and to assess whether patients received follow-up at the recommended 3-month time point. The main targets measured during this cycle were type of anticoagulant prescribed, choice of imaging modality, number of patients followed up as suggested and at what time and whether the follow-up imaging was the same as diagnosis, and any side effects or complications experienced during this period (ie, thromboembolic events, clinically significant bleeding). These results were presented at the departmental cardiology governance meeting at LTHT to highlight the improvement in practice and sustainable change.

## Results

### PDSA cycle 1

Between 1 December 2012 and 30 June 2018, a total of 84 patients were diagnosed with LV thrombus and of these 62 (74%) received VKA and 22 (26%) received DOAC. Overall, 75 (89%) underwent repeat cardiac imaging after a mean interval from diagnosis of 233±251 days and was similar comparing those who received VKA and DOAC. The same imaging modality was used for 41 (55%) patients and the mean duration of anticoagulation was 677±568 days, similar between VKA and DOAC. There was one episode of stroke and one other thromboembolic event, both of which occurred in patients receiving VKA. There were six episodes of clinically significant bleeding, all of which occurred in patients receiving VKA. We interpreted these data as demonstrating that treatment with a DOAC seemed to be similarly effective compared with VKA, with no adverse safety signal.

### PDSA cycle 2

**Figure 1:**
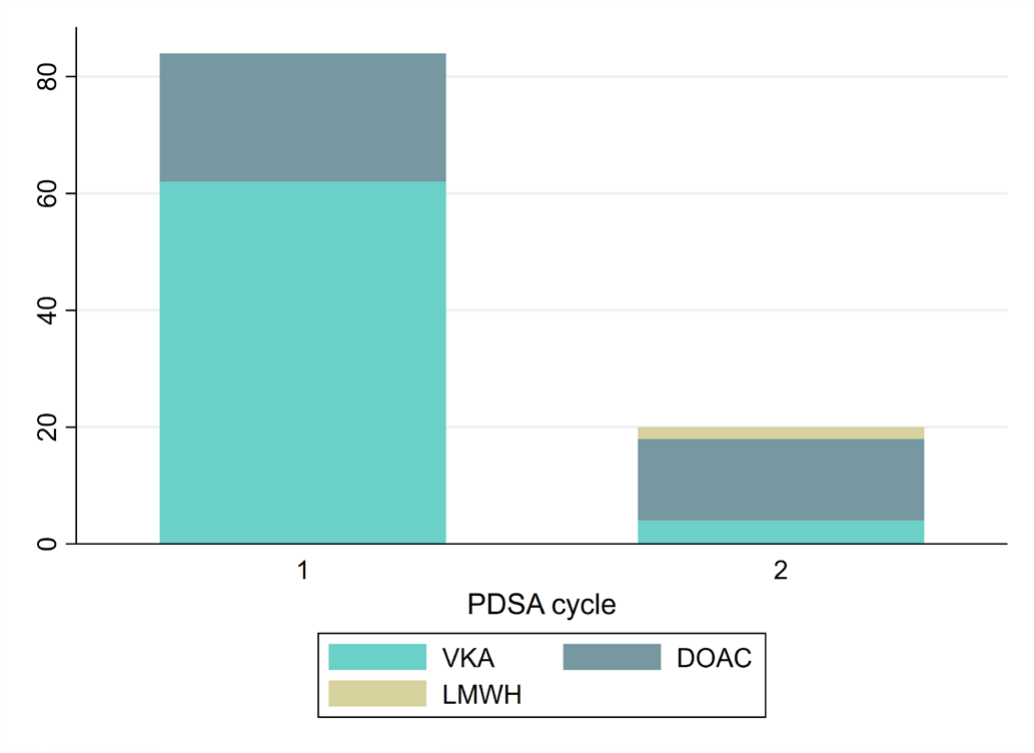
Number of patients receiving either vitamin K antagonists (VKA), direct oral anticoagulants (DOAC) or low-molecular-weight heparin (LMWH) during PDSA cycle 1 and 2. PDSA, Plan–Do–Study–Action.

Following the interventions described above, we re-evaluated our practice over a 6-month period between 1 October 2019 and 31 March 2020. Twenty patients were identified to have been newly diagnosed and managed for LV thrombus in LTHT during this period, of whom 14 (70%) received DOAC, 4 (20%) received VKA and 2 (10%) received low-molecular-weight heparin (LMWH) (figure 1).

The clinical characteristics of patients allocated to VKA, DOAC or LMWH are displayed in table 1. The majority were male (85%) and had a mean age of 60.9±11.5 years, the majority having ischaemic aetiology of heart failure (70%) and were usually receiving antiplatelet therapies alongside VKA or DOAC.

**Table 1:** Baseline characteristics of patients receiving anticoagulation for LV thrombus

	Overall (n=20)	VKA (n=4)	DOAC (n=14)	LMWH (n=2)
Demographics				
Age	60.9±11.5	63.5±5.4	58.5±12.6	70.5±9.2
Male sex	17 (85)	3 (75)	12 (85.7)	2 (100)
White-European ethnicity	18 (90)	4 (100)	12 (85.7)	2 (100)
Comorbidities				
Diabetes mellitus	6 (30)	1 (25)	3 (21)	2 (100)
Atrial fibrillation	3 (15)	1 (25)	1 (7)	1 (50)
Hypertension	5 (25)	0 (0)	4 (29)	1 (50)
Peripheral vascular disease	1 (5)	0 (0)	1 (7)	0 (0)
Stroke/TIA	2 (10)	1 (25)	1 (7)	0 (0)
PE/DVT	1 (10)	0 (0)	1 (7)	1 (50)
Smoking history	10 (50)	1 (25)	1 (7)	1 (50)
Aetiology				
Ischaemic	14 (70)	1 (25)	11 (79)	2 (100)
Dilated cardiomyopathy	3 (15)	1 (25)	2 (7)	0 (0)
Myocarditis	2 (10)	1 (25)	1 (7)	0 (0)
Antiplatelet therapy				
Aspirin	8 (40)	1 (25)	6 (30)	1 (50)
P2Y12 inhibitor	10 (50)	2 (50)	8 (57)	0 (0)

DOAC, direct oral anticoagulants; DVT, Deep vein thrombosis; LMWH, low-molecular-weight heparin; LV, left ventricular; PE, Pulmonary embolism; TIA, Transient ischaemic attack; VKA, vitamin K antagonists.

Repeat imaging modality and outcomes are displayed in table 2. Fourteen (70%) received follow-up during this period, with imaging of the same modality accounting for five (25%). The mean duration from diagnosis to follow-up was 140±61 days. We observed one episode of other embolisation, which was a pulmonary embolism occurring in the context of an individual with lung cancer while VKA had been temporarily switched to LMWH to facilitate drainage of a pleural effusion. This episode was recorded as an outcome, however, is causatively unrelated to LV thrombus. There were no cases of stroke or clinically significant bleeding observed in any of the groups.

**Table 2:** Outcomes in patients with LV thrombus

Outcomes	Overall (n=20)	VKA (n=4)	DOAC (n=14)	LMWH (n=2)
Any repeat imaging	14 (70)	2 (50)	11 (79)	1 (50)
Repeat imaging with same modality	5 (25)	1 (25)	4 (29)	0 (0)
Resolution of thrombus	7 (35)	1 (25)	6 (42)	0 (0)
Stroke	0 (0)	0 (0)	0 (0)	0 (0)
Other embolisation	1 (5)	0 (0)	0 (0)	1 (50)
Clinically significant bleeding	0 (0)	0 (0)	0 (0)	0 (0)

DOAC, direct oral anticoagulants; LMWH, low-molecular-weight heparin; LV, left ventricular; VKA, vitamin K antagonists.

Comparing the two PDSA cycles, we observed a significant change in the proportion of patients receiving DOACs for the management of LV thrombus, which increased from 26% to 70% (p<0.001), fulfilling one of our primary objectives (figure 1). The time interval to follow-up with imaging was also shorter (233±251 vs 140±61 days; p=0.10). However, the proportion of patients who underwent repeat imaging was lower in comparison to PDSA cycle 1, although the duration of follow-up was shorter.

### Lessons and limitations

Our project first aimed to assess the use of DOAC in the setting of LV thrombus. PDSA cycle 1 showed these agents were associated with similar rates of systemic thromboembolism and clinically significant bleeding compared with VKA. These findings were confirmed in a recent meta-analysis of nearly 2000 patients, including our published data, in which DOAC were non-inferior to VKA in terms for efficacy, with no significant differences in systemic thromboembolism or clinically significant bleeding.^9^ The results from the second PDSA were encouraging. We observed a transition to the use of DOAC, with no adverse safety signal and a reduction in the time interval to repeat imaging.

The overarching aim of our project was to improve the diagnosis and management of LV thrombus within LTHT with a sustainable intervention. To achieve this, we focused on developing a strategy that would be comprehensive and easily accessible. The use of PDSA cycles provided a clear plan and timeline to work towards. Communication throughout the project was essential and was more made challenging by the restrictions imposed by the COVID-19 pandemic. A key lesson learnt during the process was the importance of discussing ideas and developing them within the team. The project team consisted of consultants, junior doctors, pharmacists and a medical student which provided the perspective of a range of healthcare professionals involved in the care of these patients.

This quality improvement project has several limitations, first, the sample size obtained during PDSA cycle 1 conducted over 6 years is proportionally much smaller than that collected over the 6-month period for PDSA cycle 2 (n=84 vs 20). We believe this is due to more comprehensive screening of patients at risk with systematic echocardiography and MRI scanning in people with clinical or imaging suspicion of LV thrombus. In the primary PCI era, post-MI LV thrombus is an infrequent complication of anterior MI and of non-ischaemic cardiomyopathy, which may also explain the small sample size in our initial dataset. It should also be noted that DOAC use in atrial fibrillation was approved in 2010 so by 2012 they were starting to be used in routine care but their use in other settings such as LV thrombus was highly limited.^10^

A longer period of follow-up for PDSA cycle 2 would have also provided the opportunity to assess long-term outcomes and sustainability of the intervention, allowing for further PDSA cycles to be developed and actioned. However, additional follow-up or subsequent PDSA cycles would have included a period of the pandemic, which may have introduced biases within our data due to difficulties obtaining follow-up imaging and the transition to telephone consultations observed during this period.^11^

The results of the second PDSA cycle demonstrate that follow-up occurred at an average interval of 140±60.67 days, significantly shorter than the interval of 233±251 days in the first service evaluation. The endpoint of the data collection overlapped with the beginning of the pandemic which led to more limited access to CMR and may partially explain why only 36% of these patients were investigated with the same imaging modality. Finally, DOACs are currently unlicensed for patients with a body mass over 120 kg or patients with poor renal function (ie, creatinine clearance <30 mL/min) and our findings are therefore not generaliseable to these patients.^10^

## Conclusion

Despite the historical lack of evidence supporting their use for the treatment of LV thrombus, there is now a growing body of evidence, including our own published data, suggesting DOAC are at least as effective and safe as VKA in this setting. Optimising care of patients to reduce the risks of systemic thromboembolism with appropriate follow-up to help guide management were all considered in this project. The accessibility and simplicity of these guidelines allows the sustainability of this intervention, however further exploration of factors preventing follow-up imaging with the same modality is required. Further cycles and observation of outcomes is required to continually make amendments to the guidelines to follow the evidence base and improve efficiency of follow-up.

## Data Availability

Data are available on reasonable request.
